# Avoiding Routine Oxygen Therapy in Patients With Myocardial Infarction Saves Significant Expenditure for the Health Care System—Insights From the Randomized DETO2X-AMI Trial

**DOI:** 10.3389/fpubh.2021.711222

**Published:** 2022-01-12

**Authors:** Robin Hofmann, Tamrat Befekadu Abebe, Johan Herlitz, Stefan K. James, David Erlinge, Joakim Alfredsson, Tomas Jernberg, Thomas Kellerth, Annica Ravn-Fischer, Bertil Lindahl, Sophie Langenskiöld, DETO2X-SWEDEHEART Investigators

**Affiliations:** ^1^Division of Cardiology, Department of Clinical Science and Education, Karolinska Institutet, Södersjukhuset, Stockholm, Sweden; ^2^Department of Medical Sciences, Uppsala University, Uppsala, Sweden; ^3^Department of Health Sciences, University of Borås, Borås, Sweden; ^4^Uppsala Clinical Research Center, Uppsala University, Uppsala, Sweden; ^5^Department of Clinical Sciences, Cardiology, Lund University, Lund, Sweden; ^6^Department of Health, Medicine and Caring Sciences, Linköping University, Linköping, Sweden; ^7^Department of Cardiology, Linköping University Hospital, Linköping, Sweden; ^8^Department of Clinical Sciences, Cardiology, Karolinska Institutet, Danderyd Hospital, Stockholm, Sweden; ^9^Department of Cardiology, Faculty of Medicine and Health, Örebro University, Örebro, Sweden; ^10^Department of Molecular and Clinical Medicine, Sahlgrenska University Hospital, Gothenburg, Sweden; ^11^Department of Cardiology, University of Gothenburg, Gothenburg, Sweden

**Keywords:** myocardial infarction, health care costs and utilization, oxygen therapy, randomized clinical trial (RCT), registries (MeSH), pragmatic clinical trial

## Abstract

**Background:** Myocardial infarction (MI) occurs frequently and requires considerable health care resources. It is important to ensure that the treatments which are provided are both clinically effective and economically justifiable. Based on recent new evidence, routine oxygen therapy is no longer recommended in MI patients without hypoxemia. By using data from a nationwide randomized clinical trial, we estimated oxygen therapy related cost savings in this important clinical setting.

**Methods:** The DETermination of the role of Oxygen in suspected Acute Myocardial Infarction (DETO2X-AMI) trial randomized 6,629 patients from 35 hospitals across Sweden to oxygen at 6 L/min for 6–12 h or ambient air. Costs for drug and medical supplies, and labor were calculated per patient, for the whole study population, and for the total annual care episodes for MI in Sweden (*N* = 16,100) with 10 million inhabitants.

**Results:** Per patient, costs were estimated to 36 USD, summing up to a total cost of 119,832 USD for the whole study population allocated to oxygen treatment. Applied to the annual care episodes for MI in Sweden, costs sum up to between 514,060 and 604,777 USD. In the trial, 62 (2%) patients assigned to oxygen and 254 (8%) patients assigned to ambient air developed hypoxemia. A threshold analysis suggested that up to a cut-off of 624 USD spent for hypoxemia treatment related costs per patient, avoiding routine oxygen therapy remains cost saving.

**Conclusions:** Avoiding routine oxygen therapy in patients with suspected or confirmed MI without hypoxemia at baseline saves significant expenditure for the health care system both with regards to medical and human resources.

**Clinical Trial Registration:** ClinicalTrials.gov, identifier: NCT01787110.

## Introduction

Acute myocardial infarction (MI) occurs annually in approximately 1.5 million cases in the United States and remains one of the leading cause of mortality (1). MI was estimated to cost more than 5,000 USD per episode and remains among the most expensive diagnoses (2). In Sweden, health care costs during the first year after MI occurrence were estimated to ~15,000 USD per patient (3). Thus, as MI occurs both frequently and requires considerable health care resources, it is important to assure that the treatments which are provided are both clinically effective, and economically justifiable. Furthermore, the cost of such interventions should be evaluated at study level and overall population level to understand their full economic impact (4).

The recent randomized Determination of the role of Oxygen in suspected Acute Myocardial Infarction (DETO2X-AMI) (5) trial demonstrated definitive evidence that routine oxygen therapy provided no benefit regarding patient-reported (6) and clinical outcomes (7–11) to patients with suspected MI without hypoxemia at baseline which led to changes in guidelines (12–15) and clinical practice. No comprehensive data on therapy related costs have been available. By using unique data from a nationwide randomized clinical trial, we performed a study to estimate oxygen therapy related costs (i) on patient level, (ii) on study level, and (iii) projected on the whole annual Swedish MI population to assess potential cost-savings in this important clinical setting.

## Materials and Methods

### Study Design

The DETO2X-AMI trial was a nationwide, multicenter, open-label, registry-based randomized clinical trial (RRCT) (16) evaluating routine oxygen therapy and ambient air in normoxemic patients with suspected MI (7). The Swedish Web System for Enhancement and Development of Evidence-based Care in Heart Disease Evaluated According to Recommended Therapies (SWEDEHEART) (17) registry was used for randomization, trial procedures, and follow-up.

The study design (5), methods and primary results have been described in detail previously (7–9). The ethical review authority (Gothenburg DNR 287-12) and the medical products agency of Sweden (EudraCT 2013-002882-20) approved the trial.

The funding agencies had no access to the study data and no role in trial design, implementation, or reporting.

### Patient Population

Assessment for eligibility was carried out at first medical contact with the ambulance service, emergency department, coronary care unit or catheterization laboratory of participating hospitals. Patients were eligible if they were ≥30 years of age with typical symptoms suggestive of MI (defined as chest pain or dyspnea) for <6 h, oxygen saturation of ≥90% on pulse oximetry, and electrocardiography (ECG) changes indicating ischemia (18) or cardiac troponin levels on admission above the 99th percentile of the upper reference level.

Patients were excluded if they were hypoxemic at baseline (oxygen saturation of <90%), had continuous oxygen therapy, or cardiac arrest prior to enrollment.

### Study Procedures

#### DETO2X-AMI Trial

Eligible patients were, after initial oral informed consent, randomly assigned in an unrestricted 1:1 ratio to either oxygen therapy at 6 L/min for 6–12 h or ambient air. Randomization was performed online with a randomization module incorporated in SWEDEHEART, directly followed by initiation of allocated therapy.

Within 24 h oral consent was confirmed in writing. All patients were treated according to standard of care. Oxygen saturation was documented at the beginning and the end of the randomized treatment period. Patients received supplemental oxygen outside the protocol at the discretion of the caring physician, most commonly in cases where hypoxemia (defined as oxygen saturation <90%, including circulatory or respiratory failure) developed, which was reported separately.

#### Online Survey to Health Care Staff

In co-operation with clinical staff from the ambulance service, emergency department, cath lab, and cardiac care units, an online survey was designed to estimate the average time spent performing necessary tasks concerning oxygen therapy (Time spent to: 1. inform about oxygen treatment; 2. prepare and connect equipment for oxygen delivery; 3. adjust oxygen flow rate; 4. assisting the patient during the time of therapy; and 5. register data). The survey was distributed to the nursing staff involved in caring for patients with suspected MI of aforementioned units through the DETO2X-SWEDEHEART network and related social media using SurveyMonkey (San Mateo, CA, United States).

### Outcomes and Assumption for Cost Analyses

The primary outcome of the main trial was all-cause mortality within 1 year in the intention-to-treat population with suspected MI (7).

In the present analysis we evaluated the cost for providing supplemental oxygen to patients with suspected MI based on data from four sources:

1) Patient-related data were captured from the DETO2X-AMI trial database.
2) Staff costs attributed to time spent with individual patient care was captured from the survey (Supplementary Table A1). The average estimated time spent was multiplied with the average Swedish salary for respective employee including social security benefit (19).
3) Therapy related costs (cost of drug and medical supplies) were taken from official prices lists from the five largest regions in Sweden (Supplementary Table A2).
4) To estimate annual costs on a national level, data on the incidence and type of myocardial infarction from the SWEDEHEART annual report were used (20).

As expected, a higher proportion of patients in the ambient-air group, especially with ST-elevation MI (STEMI), developed hypoxemia (7, 8), and potential costs for oxygen treatment in such cases had to be taken into account. As data on oxygen therapy outside the protocol limits only were binary (yes/no) without specifics in cause or measures (e.g., type of mask, non-invasive or invasive ventilation, flow rate, duration of therapy), we performed a threshold analysis in which the cost per patient for hypoxemia treatment was varied to understand at which level the total costs become equal, e.g., when total costs for hypoxemia treatment in the ambient-air group exceed the total costs of routine oxygen therapy in the oxygen group. For all threshold analyses, the minimum hypoxemia treatment cost assumed in the calculation was the average cost for routine oxygen therapy per patient in the DETO2X-AMI trial.

### Secondary Analyses

First, we stratified the analysis into STEMI and Non-STEMI (NSTEMI) based on the discharge diagnoses in the SWEDEHEART registry. Cost calculations were performed in a similar manner as described above.

Second, we aimed to generalize our findings from the DETO2X trial to the general Swedish population. Unfortunately, the SWEDEHEART registry routinely covers only patients with confirmed MI, so the exact number of patients presenting with suspected MI is unknown. Moreover, baseline oxygen saturation is not recorded in the registry. Consequently, no data exist on the proportion of patients presenting with hypoxemia at baseline. In the DETO2X trial, these patients were excluded, and the pragmatic trial design did not encompass a screening log. To compensate for these limitations, we analyzed patients with confirmed MI (*N* = 16,100) based on the SWEDEHEART annual report 2015 (20) and varied the excluded proportion of patients with hypoxemia at baseline from 0 to 15%. In all cost analyses, we used the consumer price index for the year 2019. Costs were converted from Swedish Krona (SEK) to United States Dollar (USD) by the average conversion rate for 2019 of 0.11.

### Statistical Analysis

Survey findings were presented as median time [interquartile range (IQR)] to perform tasks stratified by participants workplace. Costs related to oxygen therapy were presented as average and total cost. For cost variables that had missing data, the average cost for the specific variables was imputed using simple mean imputation. Descriptive statistics of the survey and patient background characteristic was performed with R version 4.0.3 and all cost-analyses were executed using Microsoft Excel 2016.

## Results

### Study Population

Thirty-five of Sweden's sixty-nine hospitals with acute cardiac care facilities participated in the trial. Between April 13th, 2013 and December 30th, 2015, 6,629 patients (median age 68, 31% female) with suspected myocardial infarction were enrolled, 3,311 patients (50%) were allocated to oxygen and 3,318 patients (50%) were allocated to ambient air.

**Figure 1:**
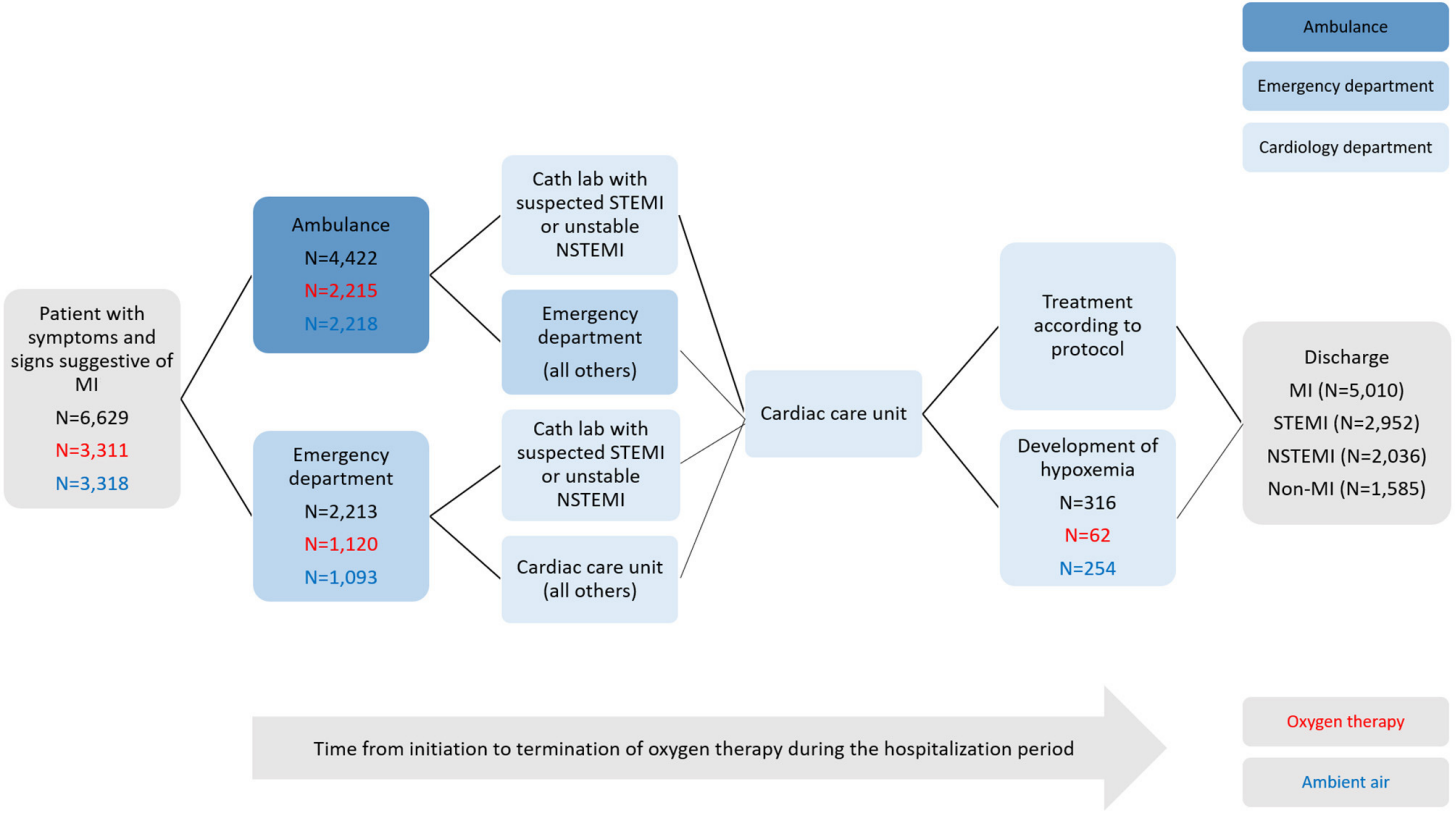
Study flow chart. Eligible patients presenting to the ambulance service, emergency departments, or cardiology department (cath lab or cardiac care units) of participating hospitals with suspected myocardial infarction were evaluated for inclusion. Shown are the numbers of patients who were enrolled in the main study, randomly assigned to a study group (in black: total count; in red allocated to oxygen therapy; in blue allocated to ambient air), treated according to protocol or developed hypoxemia, and discharge diagnoses.

Overall, 4,422 patients (67%) were transported by ambulance, 2,213 patients (33%) presented directly to the emergency department. At discharge, 5,010 (76%) patients received a primary diagnosis of MI [2,952 (59%) STEMI; 2,058 (41%) NSTEMI] (Figure 1).

In total, 316 patients (5%) received supplemental oxygen outside the protocol due to the development of hypoxemia, including 62 (2%) patients assigned to oxygen and 254 (8%) patients assigned to ambient air (*P* <0.001). However, the median duration of hospital stay was similar between the randomized groups (Table 1).

**Table 1:** Patient baseline and trial specific characteristics in the DETO2X-AMI study*.

	**Oxygen** **(*N* = 3,311)**	**Ambient air** **(*N* = 3,318)**
**Demographics – no. (%)**		
Age – years, median (IQR)	68.0 (59.0–76.0)	68.0 (59.0–76.0)
Male sex	2,264 (68.4)	2,342 (70.6)
**Causes of admission**		
Chest pain	3,123 (94.3)	3,120 (94.0)
Dyspnea	61 (1.9)	77 (2.3)
**Presentation**		
Time from symptom onset to randomization, minutes, median (IQR)	245.0 (135.0–450.0)	250 (134.0–458.0)
Ambulance transportation – no. (%)	2,215 (66.9)	2,218 (66.8)
Oxygen saturation at baseline – %, median (IQR)	97 (95–98)	97 (95–98)
**Trial procedural data**		
Duration of oxygen therapy hours, median (IQR)	11.64 (6.03–12.02)	
Received oxygen outside the protocol due to the development of hypoxemia^†^	62 (1.9)	254 (7.7)
Oxygen saturation at end of treatment Period^†^ – %, median (IQR)	99 (97–100)	97 (95–98)
Duration of hospital stay – days, median (range)	3.0 (0–68)	3.0 (0–95)
Final diagnoses – no. (%) (ICD codes)**		
MI (I.21 + I.22)	2,485 (75.1)	2,525 (76.1)
STEMI	1,431 (43.2)	1,521 (45.8)
Non-MI	1,043 (31.5)	993 (30.0)

*Plus-minus values are means ± SD. There were no significant differences in baseline characteristics between oxygen group and the ambient air group except as otherwise noted.

**Final diagnoses according to the International Classification of Diseases 10th revision (ICD-10).

*MI denotes myocardial infarction.*

*^†^P < 0.05 for the comparison between the oxygen group and the ambient air group*.

### Survey

Between July 1st, 2020 and September 16th, 2020, the online survey was available, and we received 313 replies, of which 180 were complete. Due to the distribution within the network and social media, we do not know the actual number of recipients of the survey, and thus cannot report the response rate. The median time spent related to oxygen therapy was 6 (IQR 5–10) min, 11 (IQR 6–21) min, and 9 (IQR 7–19) min from nurses from the ambulance service, emergency department, and cardiology department (cath lab and cardiac care unit).

### Cost Analysis

#### Total Study Population

The median duration of oxygen therapy was 11.6 h, with a median oxygen saturation of 99% in patients assigned to oxygen and 97% in patients assigned to air at the end of the treatment period (*P* < 0.001) (Table 1).

Costs for oxygen treatment per patient treated according to protocol were calculated to 36 USD, of which 22 USD were attributed to cost for drug and medical supplies, and 14 USD to labor costs. On study level, the corresponding figures were 119,832 USD in total, 72,972 USD, and 46,860 USD, respectively (Table 2).

**Table 2:** Total calculated cost related to oxygen therapy including cost of a drug, medical supplies, and staff per patient and care episode for patients with suspected MI.

	**Cost (in USD)**	
**Direct costs**	**Average** **per patient**	**Total per** **study population**
Oxygen therapy	11.55	38,259
Mobile tank rental*	0.01	39
Central tank rental**	1.29	4,273
Mask***	5.66	18,738
Extension cord	3.41	11,292
Connector	0.11	371
Nurse - ambulance service	2.84	8,607
Nurse - emergency department	4.87	16,119
Nurse – cardiology department	6.69	22,135
Total	36.43	119,832

*USD, United States Dollar.*

*Small tank rental cost: 0.20 USD per day which is equivalent to around 0.01 USD per hour.

**Big tank rental cost: 130 USD per month which is equivalent to around 4.30 USD per day and ~0.20 USD per hour.

***Mask cost is the average of Hudson mask (with or without reservoir) and open face mask.

**Figure 2:**
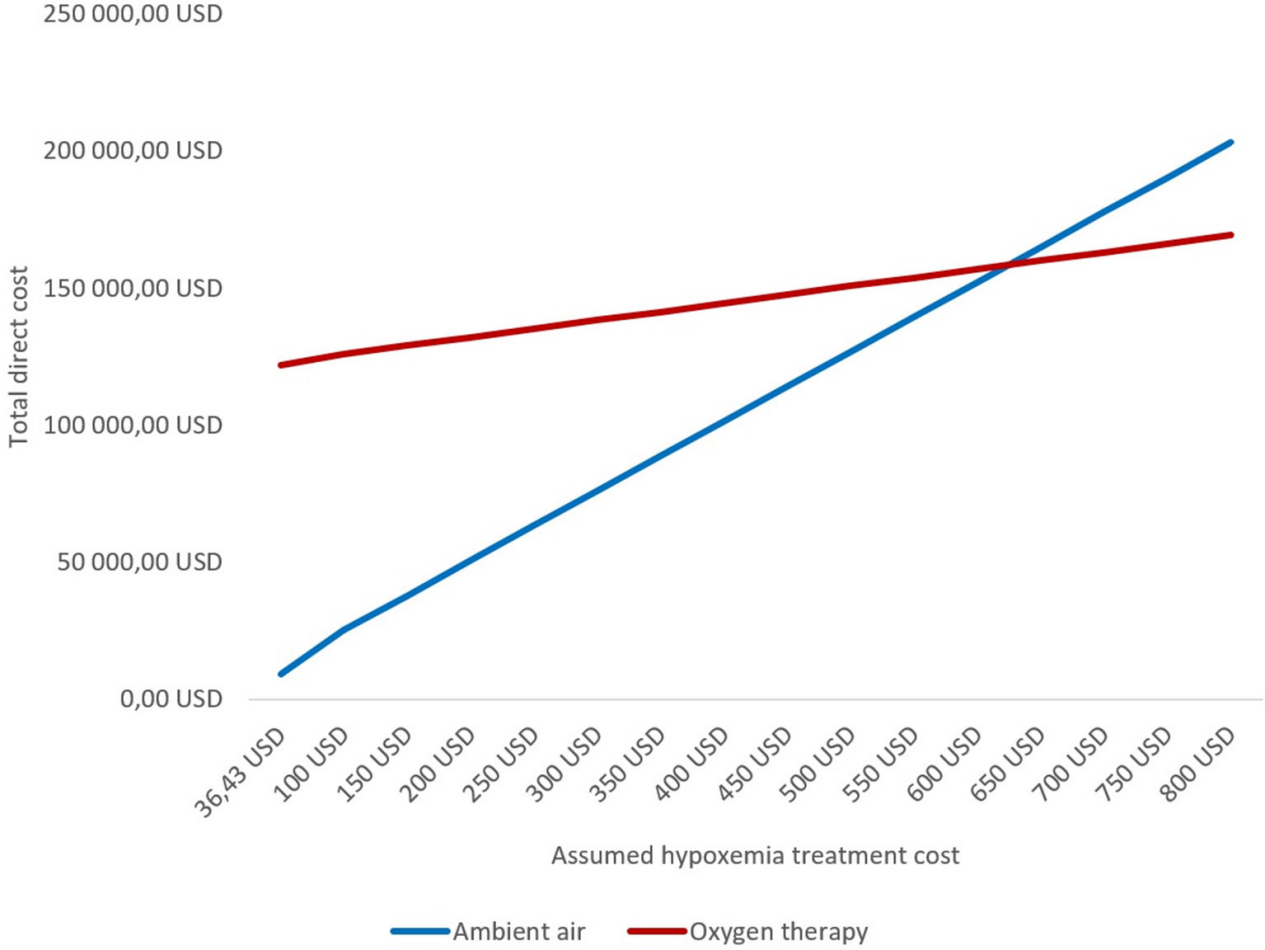
Threshold analysis of total direct cost by varying treatment costs for patients with suspected MI who develop hypoxemia.

In the threshold analysis, avoiding routine oxygen therapy remained cost saving if <624 USD were spent for hypoxemia treatment per patient (Figure 2).

#### Subgroup Analyses

##### STEMI/NSTEMI

Costs for oxygen treatment in the STEMI subgroup were calculated to 51,198 USD (36 USD per patient) compared to 38,548 USD (37 USD per patient) in patients with NSTEMI with a similar distribution of cost for drug and medical supplies, and labor costs (Supplementary Tables A3, A4).

The proportion of patients who developed hypoxemia differed between MI subtypes. In patients with STEMI, hypoxemia occurred in 236 (8%) in total, in 44 (3%) assigned to oxygen, in 192 (13%) assigned to ambient air. However, the median duration of hospital stay [3 (range 0–68) days] remained unchanged in patients with STEMI compared to the overall population (8). In patients with NSTEMI, hypoxemia occurred in 38 (1.9%) in total, in 11 (1.1%) assigned to oxygen, in 27 (2.7%) assigned to ambient air. In the threshold analysis, avoiding routine oxygen therapy remained cost saving up to 330 USD and 2,409 USD in patients with STEMI and NSTEMI, respectively (Supplementary Figures A1, A2).

#### Annual Oxygen Therapy Related Costs Calculated for the Entire MI Population in Sweden

**Figure 3:**
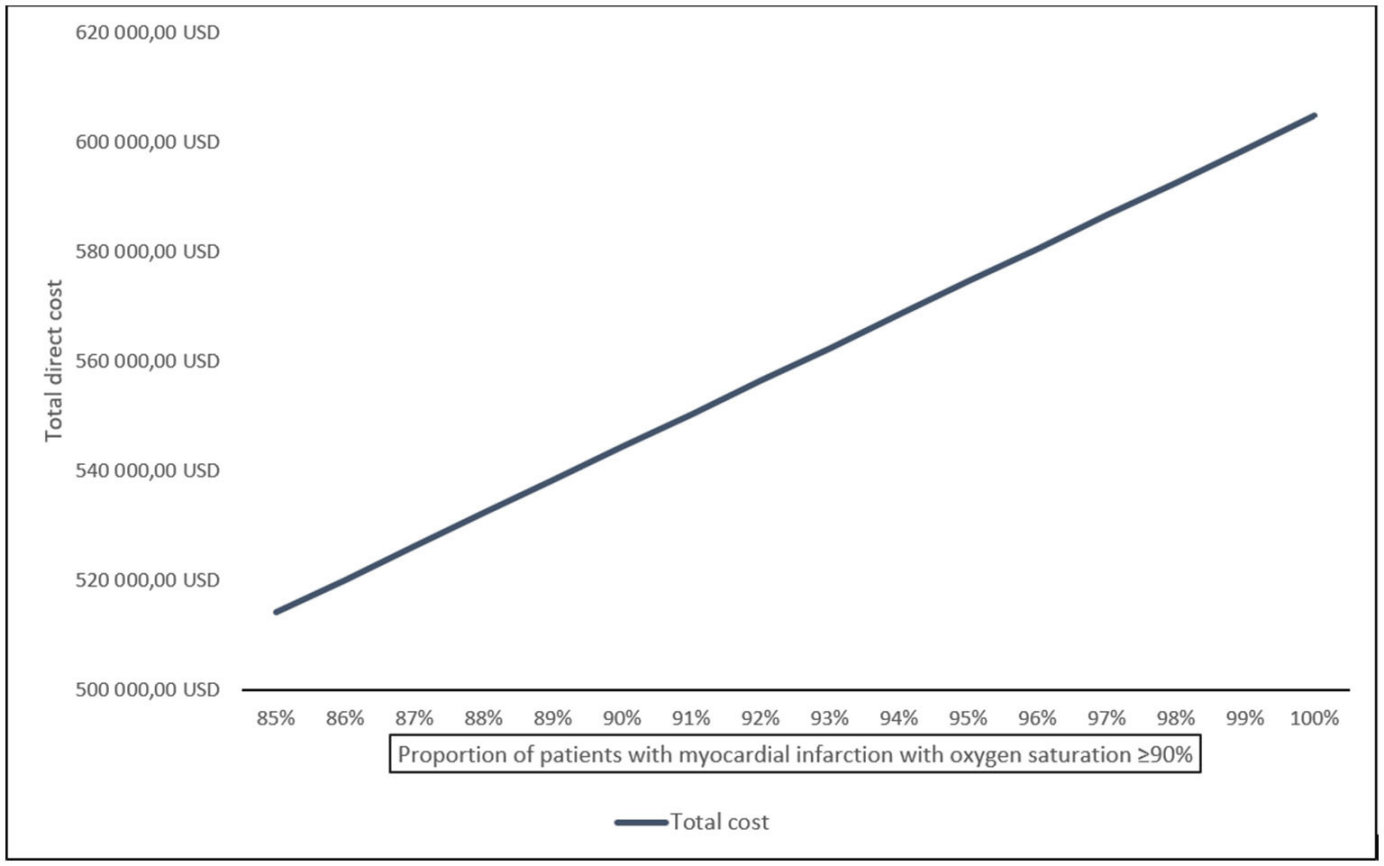
Estimated total potential cost saving of oxygen therapy for patient with confirmed MI in Sweden annually, displayed by proportion of patients with oxygen saturation ≥ 90% at baseline.

Disregarding the potential development of hypoxemia, the total potential annual cost saving for the population of 16,600 care episodes with MI were estimated between 514,060 USD (15% hypoxemic patients at baseline) and 604,777 USD (0% hypoxemic patients at baseline) (Figure 3; Supplementary Table A5).

## Discussion

Oxygen therapy has been a cornerstone of supportive care in MI for decades (21), however, guidelines and clinical practice have recently changed based on new evidence of a lack of benefit of routine supplemental oxygen therapy in patients with acute MI who have normal oxygen saturation at baseline (12–15). In this report, we present novel data on cost calculations based on the nationwide randomized DETO2X trial (5, 7), and potential cost savings from a national perspective.

Per patient enrolled in the DETO2X-AMI trial, routine oxygen therapy for MI was calculated to cost 36 USD including cost for drug and medical supplies, and labor, summing up to a total cost of 119,832 USD for the study population allocated to oxygen treatment. Applied to the annual care episodes for MI in Sweden (disregarding potential hypoxemia treatment), costs sum up to between 514,060 and 604,777 USD. As some patients will develop hypoxemia, we simulated the hypoxemia treatment cost in a threshold analysis based on the incidence of hypoxemia in the ambient-air group resulting in a theoretical cut-off at around 630 USD per patient. In other words, if the cost for treating one patient with hypoxemia was below that theoretical threshold, withholding routine oxygen remains the more cost-effective approach despite a higher rate of patients who develop hypoxemia in the ambient-air group. Based on the higher proportion of patients with STEMI developing hypoxemia, the threshold in those individuals was lower at 356 USD. Notably, the duration of hospital stay was unchanged regardless of randomized therapy or MI subtype (7, 8). This may indicate that most of these patients were treated sufficiently with standard of care similar to the oxygen strategy in the trial (oxygen delivered by open face mask at 6 L/min for up to 12 h) with a corresponding cost of around 36 USD. Thus, routine oxygen therapy remains unjustified from an economic perspective.

In light of the 5,000 USD for a care episode of MI (2) the average total estimated cost of 36 USD per patient for oxygen therapy may appear small for a health care intervention. However, when widening the perspective to the healthcare system on an annual basis, the impact becomes much larger, e.g., for Sweden projected to more than half a million USD annually. It is important to remember that this calculation is based on patients with confirmed MI. As the diagnosis of MI is often not clear at presentation, the number of patients with suspected MI, and accordingly the total cost of care, is much higher. In the DETO2X trial, 24% of patients were discharged with a Non-MI diagnosis (22) whereas in other cohorts, the proportion of suspected to confirmed MI was substantially greater (23, 24). In a global perspective, the number of care episodes for suspected MI are staggeringly high (25) and constitute a considerable burden to the strained health care systems. Moreover, as we have learned during the current pandemic, oxygen is a valuable but limited resource which should be saved to those in greatest need and with clear medical and/or economical indication (26).

It is challenging to put these findings into perspective as limited data on the cost of oxygen therapy in the clinical setting exist. However, the Thrombus Aspiration in ST-Elevation Myocardial infarction (TASTE) trial evaluating the effect of routine thrombus aspiration on death in patients with STEMI, was performed in a similar clinical context and utilized the same framework and RRCT design as our trial (27). It was shown that thrombus aspiration prior to angioplasty was not beneficial in terms of mortality (28) and, consequently, the nationwide use of thrombus aspiration in Sweden dropped from 40 to 12% (29). Similar to the current cost study from DETO2X, the investigators demonstrated an annual cost reduction of about 150,000 USD for the health care system (29).

Another important aspect in the setting of acute MI is the time health care personnel spends on specific measures, in particular in the setting of STEMI where timely reperfusion is a class 1A recommendation (30). Recently, it was shown that time from symptom onset to revascularization is strongly correlated with infarct size and prognosis (31). In our survey, the ambulance staff estimated to have spent in average 6 min on oxygen therapy related tasks. Naturally, other tasks and/or transportation were performed simultaneously during those minutes, however, prioritization and time efficiency remain crucial, and any unnecessary delay should be omitted (31, 32).

The RRCT concept has emerged as a useful trial design to provide evidence on the comparative efficacy and safety of different therapeutic strategies used in routine clinical practice (33). Recently, the TASTE investigators provided an example on the nationwide impact of the trial results in terms of implementation, cost effectiveness and in relation to trial costs (29). On top of the cost minimization on national level described above, they reported an average cost of 400,000 USD to conduct the trial, an estimated 10-fold reduction compared to costs of a similar conventional RCT. The DETO2X trial cost were slightly higher, at around 800,000 USD, due to the more complex research environment of the ambulance service and ED on top of cardiac care facilities. Nevertheless, we can hereby provide another example of the value of a pragmatic clinical trial in optimizing the quality of care given to patients while avoiding unnecessary medical expenditure for the health-care system. Thanks to recent comprehensive evidence, oxygen treatment in the setting of MI is by today recommended only in patients with hypoxemia (defined as oxygen saturation <90%) (12–15). However, conclusive data on the utility of oxygen therapy is still lacking in other important clinical scenarios such as heart failure, after cardiac arrest, or stroke, both from a clinical, but not in the least from a cost-effectiveness perspective for the health care system. Future randomized trials conducted in the context of clinical practice may be a good tool to close these gaps of knowledge.

General and conceptual limitations to the DETO2X study have been described in detail previously (7). First, calculations presented in the current analysis are based on the Swedish standard of care and population, and the Swedish public health care system, which may not directly apply for other health care systems. Second, the time dedicated by the health care staff to oxygen related tasks was not documented during the trial and information was captured retrospectively by means of a survey. We lack data to calculate the response rate overall, and there was a degree of missingness throughout the survey. Third, we lacked detailed information on the proportion of MI patients presenting with hypoxemia, and data on specific, potentially costly measures to treat this condition. Therefore, we conducted threshold analyses varying proportions and cost estimates.

## Conclusions

Avoiding routine oxygen therapy in patients with suspected or confirmed MI without hypoxemia at baseline saves significant expenditure for the health care system both with regards to medical- and human resources. The RRCT concept to perform pragmatic clinical trials can be utilized to evaluate care processes in daily care from a clinical and cost-benefit perspective, while simultaneously enabling efficient medical research.

## Data Availability Statement

The datasets presented in this article are not readily available because public data sharing not granted by the Ethics Committee. Requests to access the datasets should be directed to robin.hofmann@sll.se.

## Ethics Statement

The studies involving human participants were reviewed and approved by the Ethical Review Authority Gothenburg, Sweden (DNR 287-12). The patients/participants provided their written informed consent to participate in this study.

## Author Contributions

RH, TA, BL, and SL had full access to all of the data in the study and take responsibility for the integrity of the data and the accuracy of the data analysis, concept and design, drafting of the manuscript, administrative, technical, or material support, and supervision. TA and SL: statistical analysis. RH and JH: obtained funding. All authors acquisition, analysis, or interpretation of data and critical revision of the manuscript for important intellectual content.

## Funding

RH was supported by the Region Stockholm (clinical postdoctoral appointment, Grant Number K 2017-4577) and Swedish Heart-Lung Foundation (Grant Number, HLF 2018-0187). JH was supported Swedish Heart-Lung Foundation (Grant Number, HLF 20160688). The Region Stockholm and the Swedish Heart-Lung Foundation had no role in the design and conduct of the study; collection, management, analysis, and interpretation of the data; preparation, review, or approval of the manuscript; and decision to submit the manuscript for publication.

## Conflict of Interest

The authors declare that the research was conducted in the absence of any commercial or financial relationships that could be construed as a potential conflict of interest.

## Publisher's Note

All claims expressed in this article are solely those of the authors and do not necessarily represent those of their affiliated organizations, or those of the publisher, the editors and the reviewers. Any product that may be evaluated in this article, or claim that may be made by its manufacturer, is not guaranteed or endorsed by the publisher.
