# Supplementary material for: Avoiding Routine Oxygen Therapy in Patients With Myocardial Infarction Saves Significant Expenditure for the Health Care System—Insights From the Randomized DETO2X-AMI Trial
Source: Front Public Health. 2022 Jan 12;9:711222. doi: 10.3389/fpubh.2021.711222 (PMC8790120; doi:10.3389/fpubh.2021.711222)
Supplement: Supplementary file 1 [file Data_Sheet_1.PDF]

DETO2X-AMI

PARTICIPATING CENTERS AND INVESTIGATORS

Danderyd University Hospital, Stockholm, Sweden. Rickard Linder, Mattias Ekström.

Enköping Hospital, Sweden. Lena Forsman.

Gothenburg University Hospital (Sahlgrenska), Sweden. Johan Herlitz, Annica Ravn-Fischer, Elmir Omerovic, Oskar Angerås.

Gothenburg University Hospital (Östra), Sweden. Björn Hornestam.

Gävle Hospital, Sweden. Robert Kastberg, Espen Haugen.

Hallands Hospital, Halmstad, Sweden. Markus Lingman.

Hallands Hospital, Varberg, Sweden. Markus Lingman.

Härnosand Hospital, Sundsvall, Sweden. Anna Millbourn.

Kalmar Regional Hospital, Sweden. Anders Engström, Jörg Carlsson.

Karlstad Hospital, Sweden. Urban Haaga.

Karolinska University Hospital, Huddinge, Sweden. Tomas Jernberg.

Karolinska University Hospital, Solna, Sweden. John Pernow, Linda Mellbin, Dinos Verouhis.

Kiruna Hospital, Sweden. Fredrik Kjellberg.

Kristianstad Hospital, Sweden. Raluca Jumatate.

Köping Hospital, Sweden. Lennart Malmqvist, Gull-Britt Eriksson.

Lindesberg Hospital, Sweden. Thomas Kellerth.

Linköping University Hospital, Sweden. Joakim Alfredsson, Lennart Nilsson, Eva Swahn, Dimitrios Venetsanos.

Ljungby Hospital, Sweden. Carina Nilsson.

Norrlands University Hospital, Umeå, Sweden. Krister Lindmark.

Norrtälje Hospital, Sweden. Tommy Pettersson, Melvin Pourbazargan.

Nyköping Hospital, Sweden. Martin Serrander.

Ryhov Hospital, Jönköping, Sweden. Jörg Lauermann, Jan-Erik Karlsson, Neshro Barmano.

Sahlgrenska Universitetssjukhus Mölndal, Sweden. Martin Risenfors.

Skaraborgs Hospital, Lidköping, Sweden. Magnus Peterson.

Skaraborgs Hospital, Skövde, Sweden. Ylwa Wallström.

Skåne University Hospital, Lund, Sweden. David Erlinge, David Sparv, Ulf Ekelund.

Skåne University Hospital, Malmö, Sweden. David Erlinge, David Sparv.

St: Göran Hospital, Stockholm, Sweden. Marianne Erlandsson.

Södersjukhuset, Stockholm, Sweden. Robin Hofmann, Nils Witt, Mats Frick, Leif Svensson, Ellinor Berglund.

Trelleborg Hospital, Sweden. Troels Yndigegn.

Uppsala University Hospital, Sweden. Stefan James, Bertil Lindahl, Gabriel Arefalk, Bo Lagerqvist.

Vrinnevi Hospital, Norrköping, Sweden. Christofer Digerfeldt.

Växjö Hospital, Sweden. Olle Bergström.

Örebro University Hospital, Sweden. Thomas Kellerth

Örnsköldsvik Hospital. Björn Byström.
