# Supplementary material for: Avoiding Routine Oxygen Therapy in Patients With Myocardial Infarction Saves Significant Expenditure for the Health Care System—Insights From the Randomized DETO2X-AMI Trial
Source: Front Public Health. 2022 Jan 12;9:711222. doi: 10.3389/fpubh.2021.711222 (PMC8790120; doi:10.3389/fpubh.2021.711222)
Supplement: Supplementary file 2 [file Data_Sheet_2.PDF]

Supplemental appendix

To:

Avoiding routine oxygen therapy in patients with myocardial infarction saves significant expenditure for the health care system

– Insights from the randomized DETO2X-AMI trial

Robin Hofmann, M.D., Ph.D.[a]; Tamrat Befekadu Abebe*,[b]; Johan Herlitz, M.D., Ph.D.[c]; Stefan K. James, M.D., Ph.D.,[b,d]; David Erlinge, M.D., Ph.D.[e]; Joakim Alfredsson, M.D., Ph.D.;[f] Tomas Jernberg, M.D., Ph.D.[g]; Thomas Kellerth, M.D.;[h] Annica Ravn-Fischer, M.D., Ph.D.; Bertil Lindahl, M.D., Ph.D.[b,d]; and Sophie Langenskiöld, PhD,[b] for the DETO2X-SWEDEHEART Investigators*

a) Department of Clinical Science and Education, Division of Cardiology, Karolinska Institutet, Södersjukhuset, Stockholm, Sweden.
b) Department of Medical Sciences, Uppsala University, Uppsala, Sweden
c) Department of Health Sciences, University of Borås, Borås, Sweden.
d) Uppsala Clinical Research Center, Uppsala University, Uppsala, Sweden.
e) Department of Clinical Sciences, Cardiology, Lund University, Lund, Sweden.
f) Department of Health, Medicine and Caring Sciences, Linköping University, and Department of Cardiology, Linköping University hospital, Linköping, Sweden.
g) Department of Clinical Sciences, Cardiology, Karolinska Institutet, Danderyd Hospital, Stockholm, Sweden.
h) Department of Cardiology, Faculty of Medicine and Health, Örebro University, Örebro
i) Department of Molecular and Clinical Medicine and Sahlgrenska University Hospital, Department of Cardiology, University of Gothenburg, Gothenburg, Sweden

*Shared first author

Table of contents

Summary of estimated staff costs related to oxygen therapy (table A1)	3
Summary of estimated costs of oxygen and medical supplies (table A2)	4
Direct medical cost of oxygen therapy in patients with STEMI (table A3)	5
Direct medical cost of oxygen therapy in patients with NSTEMI (table A4)	6
Two-way sensitivity analysis of the cost of oxygen therapy compared to ambient air for patients with MI in Sweden (table A5)	7
Threshold analysis of total direct cost by varying treatment costs for patients with STEMI who develop hypoxemia (figure A1)	8
Threshold analysis of total direct cost by varying treatment costs for patients with NSTEMI who develop hypoxemia (figure A2)	9

Table A1: Summary of estimated time spent with tasks related to oxygen therapy

	Ambulance service	Emergency department	Cardiology department
	(n=63)	(n=31)	(n=86)
Tasks<br>minutes, median (IQR)			
Inform the patient	1 (1-2)	2 (1-5)	2 (1-5)
Prepare and connect equipment	1 (1-2)	2 (1-5)	2 (1-5)
Adjust oxygen flow rate	1 (1-1)	1 (1-3)	1 (1-2)
Assisting the patient	1 (1-1.5)	2 (1-5)	2 (1-3.75)
Register data	1 (1-2)	3 (1-5)	2 (1-4)
Average total time	6 (5-10)	11 (6-20.5)	9 (7-18.8)

IQR denotes interquartile range

Table A2: Summary of estimated costs of oxygen and medical supplies

Variable	Unit	Value
Hudson mask	USD	1.21
Hudson mask with reservoir	USD	3.83
Open face mask	USD	3.74
Extension cord	USD	3.55
Connector	USD	0.12
Cost of oxygen therapy per liter (mobile tank)	USD	0.02
Cost of oxygen therapy per liter (central tank)	USD	<0.001
Rental cost of oxygen mobile tank per hour	USD	0.01
Rental cost of oxygen central tank per hour	USD	0.18
Salary (nurses), per hour		
Ambulance service	USD	43.17
Emergency department	USD	42.19
Cardiology department	USD	46.51
Social security benefit	%	51
USD: United States Dollar		

In patients transported by ambulance, a closed Hudson mask with or without reservoir bag was used. We assumed Hudson masks with reservoir (standard for most ambulance organizations at the time) in ¾ of the cases and in ¼ Hudson masks without reservoir. Hudson masks were replaced by an open face mask which was used in all cases from hospital arrival throughout the duration of the treatment. In the ambulance service and during transportation between units at hospital, a mobile oxygen tank was used. From arrival at hospital, the oxygen

was supplied from the central tank of the hospital. Regardless of facility or type of mask, the oxygen flow rate was constant at 6L/min for the duration of the intervention.

Table A3: Total calculated cost related to oxygen therapy including cost of a drug, medical supplies, and staff per patient and care episode for patients with STEMI

	Cost (in USD)	
Direct costs	Average	Total
Oxygen therapy	12.25	17, 528
Small tank rental*	0.01	18
Big tank rental**	1.23	1,755
Mask***	6.00	8,591
Extension cord	3.41	4,880
Connector	0.11	160
Nurse - ambulance service	3.31	4,443
Nurse - emergency department	2.97	4,244
Nurse – cardiology department	6.69	9,574
Total	35.99	51,198
USD: United States Dollar		

*Small tank rental cost: 0.20 USD per day which is equivalent to around 0.01 USD per hour.

** Big tank rental cost: 130 USD per month which is equivalent to around 4.30 USD per day and approximately 0.20 USD per hour.

*** Mask cost is the average of Hudson mask (with or without reservoir) and open face mask.

Table A4: Total calculated cost related to oxygen therapy including cost of a drug, medical supplies, and staff per patient and care episode for patients with NSTEMI

	Cost (in USD)	
Direct costs	Average	Total
Oxygen therapy	11.11	11,584
Mobile tank rental*	0.01	12
Central tank rental**	1.38	1,442
Mask***	5.43	5,662
Extension cord	3.41	3,557
Connector	0.11	117
Nurse - ambulance service	2.49	2,416
Nurse - emergency department	6.50	6,782
Nurse – cardiology department	6.69	6,977
Total	37.13	38,549
USD: United States Dollar		

*Mobile tank rental cost: 0.20 USD per day which is equivalent to around 0.01 USD per hour.

** Cedntral tank rental cost: 130 USD per month which is equivalent to around 4.30 USD per day and approximately 0.20 USD per hour.

*** Mask cost is the average of Hudson mask (with or without reservoir) and open face mask.

Table A5: Two-way sensitivity analysis of the cost of oxygen therapy compared to ambient air for patients with MI in Sweden

Hypoxemia treatment cost	Probability of oxygen saturation >90% on admission			
	85%	90%	95%	100%
0.00 USD	514 060 USD	544 299 USD	574 538 USD	604 777 USD
49.20 USD	484 334 USD	512 824 USD	541 314 USD	569 805 USD
100.00 USD	432 467 USD	457 906 USD	483 345 USD	508 785 USD
200.00 USD	350 874 USD	371 513 USD	392 153 USD	412 792 USD
300.00 USD	269 280 USD	285 120 USD	300 960 USD	316 800 USD
400.00 USD	187 687 USD	198 727 USD	209 767 USD	220 808 USD
500.00 USD	106 093 USD	112 334 USD	118 575 USD	124 816 USD
600.00 USD	24 500 USD	25 941 USD	27 382 USD	28 823 USD
630.03 USD	0,00 USD	0,00 USD	0,00 USD	0,00 USD
700.00 USD	-57 094 USD	-60 452 USD	-63 811 USD	-67 169 USD
800.00 USD	-138 687 USD	-146 845 USD	-155 003 USD	-163 161 USD
900.00 USD	-220 281 USD	-233 238 USD	-246 196 USD	-259 154USD
1 000.00 USD	-301 874 USD	-319 631 USD	-337 389 USD	-355 146 USD

A two-way sensitivity analysis was executed to estimate annual costs for the entire Swedish MI population by varying the proportion between 85%-100% of patients with oxygen saturation ≥ 90% at baseline, and the cost of treating hypoxemia by means of a threshold analysis.

Figure A1: Threshold analysis of total direct cost by varying treatment costs for patients with STEMI who develop hypoxemia

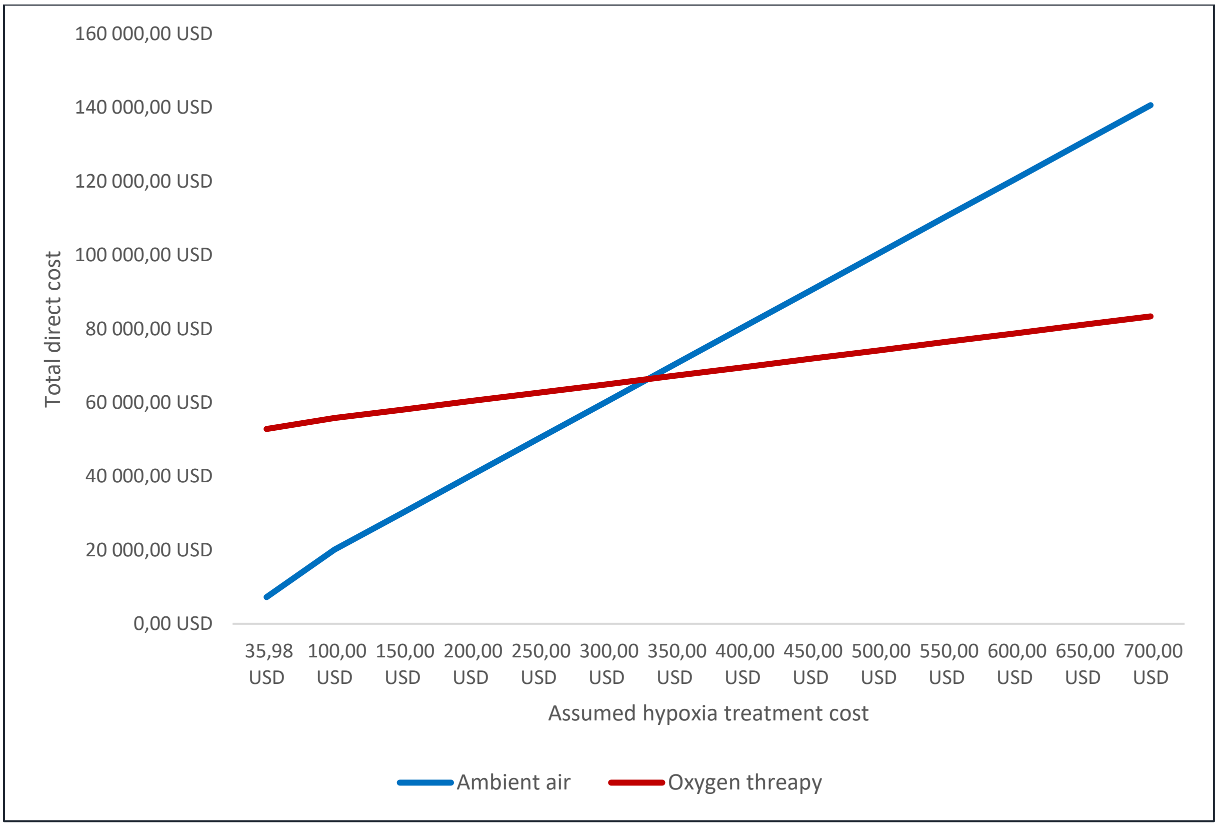

Threshold analysis to display the impact of the potential costs on the cost-benefit ratio per treatment group. The threshold was defined as the average total cost for oxygen treatment in patients in the ambient-air group who develop hypoxemia at which the cost for routine oxygen treatment per patient becomes the more cost-effective approach. The minimum hypoxia treatment cost assumed in the calculation was the average cost for routine oxygen therapy per ST-elevation myocardial infarction (STEMI) patient from the DETO2X-AMI trial (36 USD).

In the threshold analysis, routine oxygen therapy becomes cost saving at 330 USD in patients with STEMI.

Figure A2: Threshold analysis of total direct cost by varying treatment costs for patients with NSTEMI who develop hypoxemia

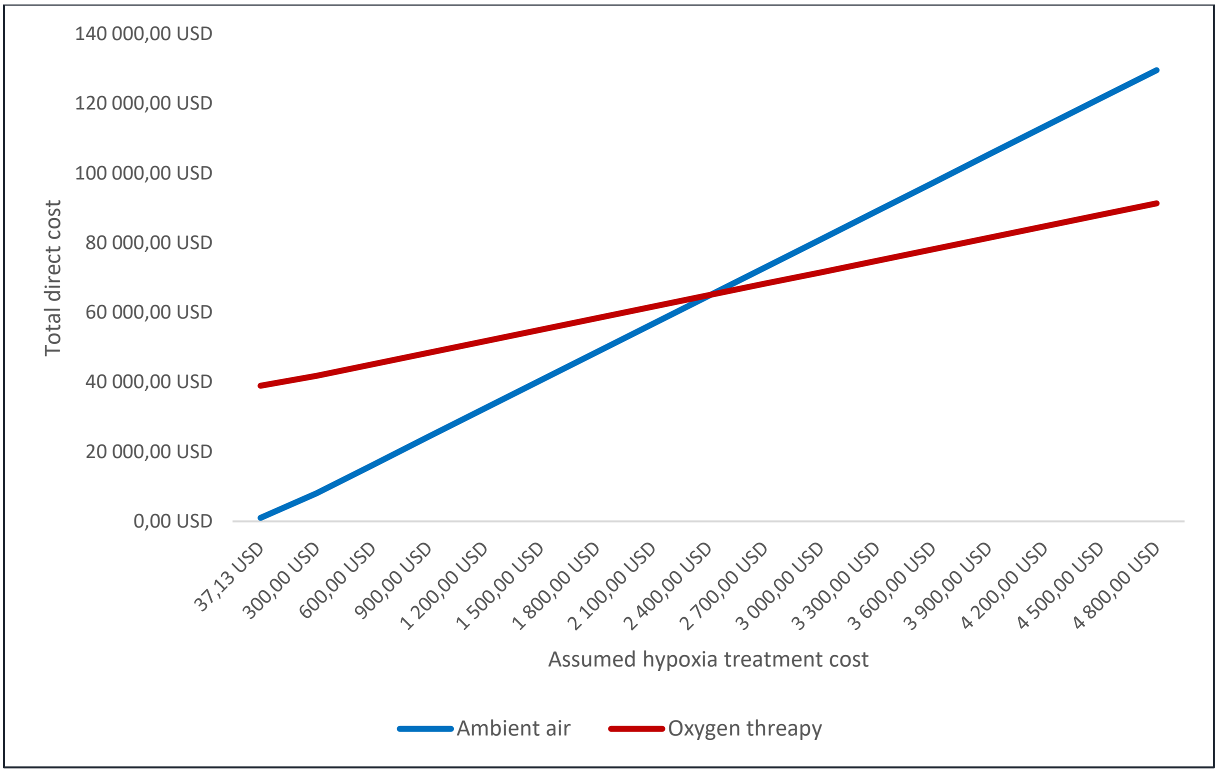

Threshold analysis to display the impact of the potential costs on the cost-benefit ratio per treatment group. The threshold was defined as the average total cost for oxygen treatment in patients in the ambient-air group who develop hypoxemia at which the cost for routine oxygen treatment per patient becomes the more cost-effective approach. The minimum hypoxia treatment cost assumed in the calculation was the average cost for routine oxygen therapy per Non-ST-elevation myocardial infarction (NSTEMI) patient from the DETO2X-AMI trial (37 USD).

In the threshold analysis, routine oxygen therapy becomes cost saving at 2,409 USD in patients with NSTEMI.
